# Misaligning Cascaded Liquid Crystal Elements for Continuously Tunable Axial Spin‐Dependent Splitting

**DOI:** 10.1002/nap2.70270

**Published:** 2026-08-29

**Authors:** Yougang Ke, Xinhuai Gu, Linzhou Zeng, Zhangming Hong, Jingqing Yu, Yingqun Xiao, Xinxing Zhou, Jin Li

**Affiliations:** ^1^ School of Information Science and Engineering Hunan Institute of Science and Technology Yueyang China; ^2^ Key Laboratory of Low‐Dimensional Quantum Structures and Quantum Control of Ministry of Education School of Physics and Electronics Hunan Normal University Changsha China; ^3^ School of Instrumentation and Optoelectronic Engineering Beihang University Beijing China

**Keywords:** axial spin splitting, cascaded liquid crystal elements, continuous tunability, photonic spin Hall effect

## Abstract

Achieving continuous tunability and spin‐multiplexed functionality is essential for depth‐resolved imaging, yet existing approaches suffer from fixed focal lengths, transverse‐plane splitting, or inability to acquire two depth planes simultaneously. Here, we propose and experimentally demonstrate a cascaded liquid crystal Pancharatnam–Berry phase system. It comprises two phase elements and a classic convex lens, which enables continuously tunable axial spin‐dependent splitting via controlled lateral misalignment. A relative in‐plane displacement generates a spin‐dependent quadratic phase, yielding equivalent focal powers linear with displacement and opposite signs for orthogonal spins. This mechanism continuously tunes the focal lengths of left and right circularly polarized light, producing two separate foci at distinct axial positions for parallel depth acquisition. Reversing the displacement sign dynamically interchanges the two foci. Leveraging this capability, we demonstrate multiplane imaging in a single exposure, eliminating mechanical axial scanning required in traditional microscopes. An analytical model is established to quantitatively link the lateral misalignment to the two focal positions. This work establishes misalignment‐enabled axial spin splitting as a compact and efficient platform for depth‐resolved and spin‐dependent optical systems.

## Graphical abstract

Continuously tunable axial spin splitting is achieved by controlling the lateral misalignment of cascaded Pancharatnam‐Berry phase liquid crystal elements. The displacement generates a spin‐dependent quadratic phase, enabling linear, sign‐reversible focal adjustment and dynamic focus interchange for orthogonal circular polarizations. This compact platform achieves single‐exposure parallel multiplane imaging, completely eliminating the need for traditional axial mechanical scanning.

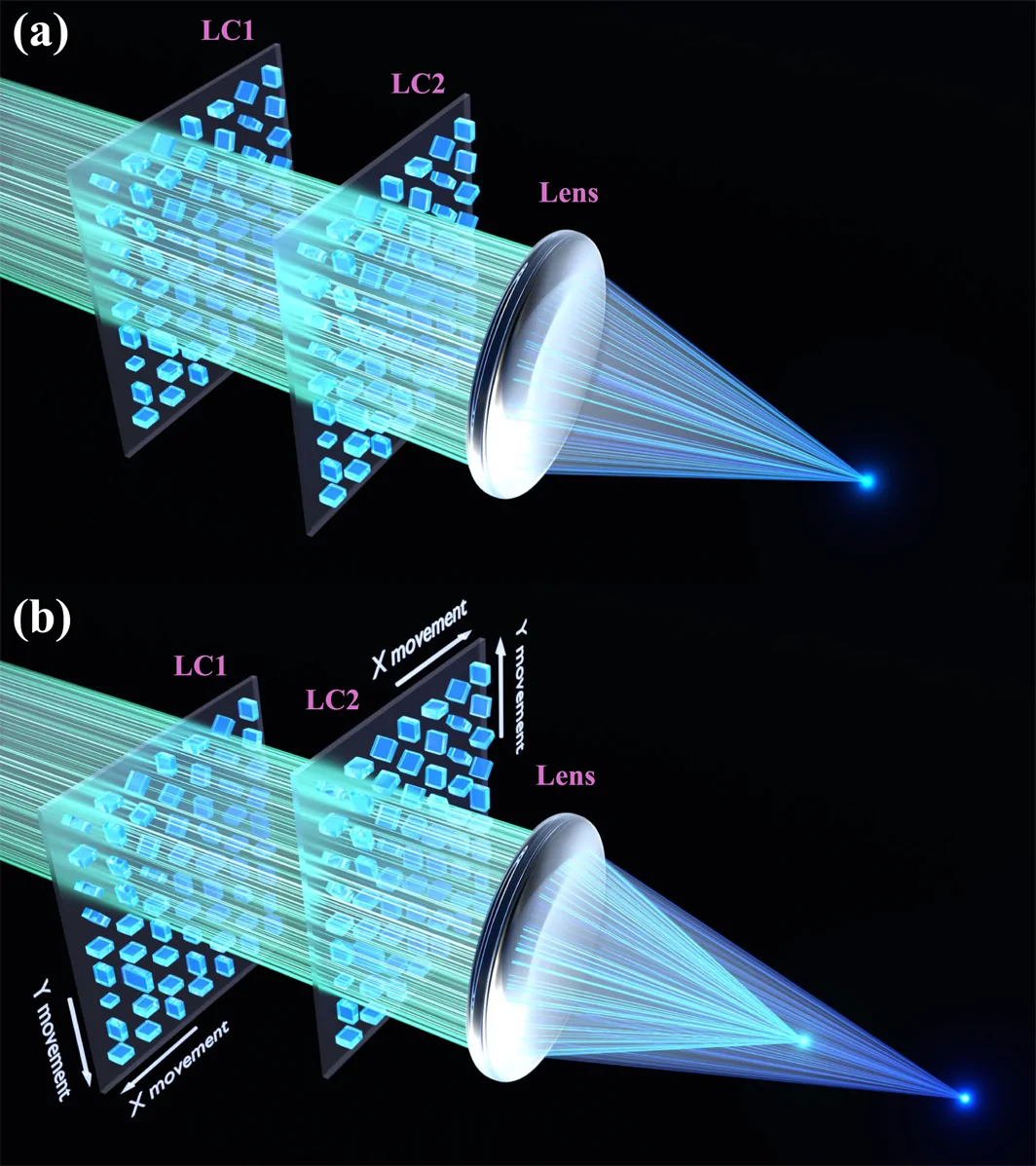

## 1 Introduction

The photonic spin Hall effect (PSHE), a fundamental spin–orbit coupling phenomenon in optics, manifests as a spin‐dependent transverse shift of a light beam upon propagation through inhomogeneous media or at interfaces [1, 2, 3]. Owing to the extremely small photon momentum, this shift is inherently weak and was initially difficult to observe without weak measurement techniques or multiple reflections [4]. The emergence of metasurfaces has dramatically altered this situation [5]. Through the design of subwavelength structure [6, 7, 8], metasurfaces strongly enhance the spin–orbit interaction and generate sizable spin‐dependent geometric phases [9, 10], enabling clear experimental observation of the PSHE [2]. These advances have opened up a broad range of applications, including precision metrology [11], optical information processing [12], chiral sensing [13], optical analog computing [14, 15, 16, 17, 18], and spin‐based photonic devices [19].

Spin‐dependent splitting has been successfully demonstrated in the transverse plane using Pancharatnam–Berry (PB) phase elements [20]. Extending this capability to the axial dimension is far more compelling for computational imaging [21, 22], yet existing axial approaches each exhibit certain limitations. For example, Ke et al. combined a PB phase lens with a dynamic phase lens to achieve spin‐selective focusing, but the design was static and lacked continuous varifocal capability [23]. An electrically tunable bifocal metalens was reported by Badloe et al., offering fast switching between spin states; nevertheless, the two focal distances are rigidly fixed [24]. Xu et al. produced two discrete foci via block‐wise multiplexing, again without any tunability [25]. Besides, Lin et al. introduced a rotation‐based varifocal meta‐lens, but its polarization‐independent operation yields only a single sharp image at any instant, preventing the parallel acquisition of multiple depth planes [26]. Li et al. achieved axial focal‐length tuning through a twisted bilayer meta‐device; yet, only right circularly polarized (RCP) light is focused, whereas left circularly polarized (LCP) light remains divergent, thus failing to provide true spin‐multiplexed dual‐focus operation [27]. Zheng et al. reviewed the application of integrated metasurfaces for on‐chip optical field manipulation in semiconductor optoelectronic devices [28], and also demonstrated broadband vectorial holography from visible to deep‐ultraviolet using diamond metasurfaces [29]. In contrast, liquid crystal (LC) devices offer an alternative reconfigurable platform, with advantages such as large aperture fabrication and relatively simple preparation, complementing metasurface schemes. Consequently, a system that simultaneously delivers continuous axial tuning, two independent spin‐channel foci, and parallel imaging of distinct depth planes has remained elusive.

Here, we address this gap by demonstrating a cascaded liquid–crystal system that leverages lateral misalignment between two PB‐phase LC elements with cubic phase profiles. A controlled relative displacement breaks the initial symmetry and produces a quadratic phase term whose magnitude is proportional to the displacement and whose sign reverses for the two circular polarization states. This generates a spin‐dependent equivalent focal power, enabling continuous tuning of the LCP and RCP focal positions along the optical axis. Importantly, the axial splitting distance can be varied from positive, through zero, to negative values, so that the LCP and RCP foci can be positioned on opposite sides of a reference plane and their relative order dynamically exchanged. The system thus simultaneously provides two spin‐separated foci at different depths, allowing parallel capture of proximal and distal image information within a single measurement.

Our experimental configuration integrates the two cascaded LC PB elements with a traditional convex lens, as illustrated in Figure 1. In the aligned state, the device behaves as a conventional lens and yields a single focus. Upon introducing a diagonal lateral displacement, the LCP and RCP components separate along the optical axis into two distinct foci, and reversing the displacement interchanges their axial positions. We continuously tune the focal lengths over a displacement range of ±0.6 mm, preserving high imaging quality throughout. Compared with existing designs based on rotation, electrical switching, or fixed multi‐foci configurations, our misalignment‐based approach offers several key advantages: (i) continuous, reversible, and sign‐controllable axial spin splitting; (ii) true parallel spin‐dependent dual‐plane imaging with both channels simultaneously in focus; and (iii) high imaging fidelity retained across the entire tuning range. These results establish lateral‐misalignment‐enabled axial spin splitting as a versatile tool for depth‐resolved imaging, spin‐dependent information processing, and reconfigurable photonic systems.

**FIGURE 1:**
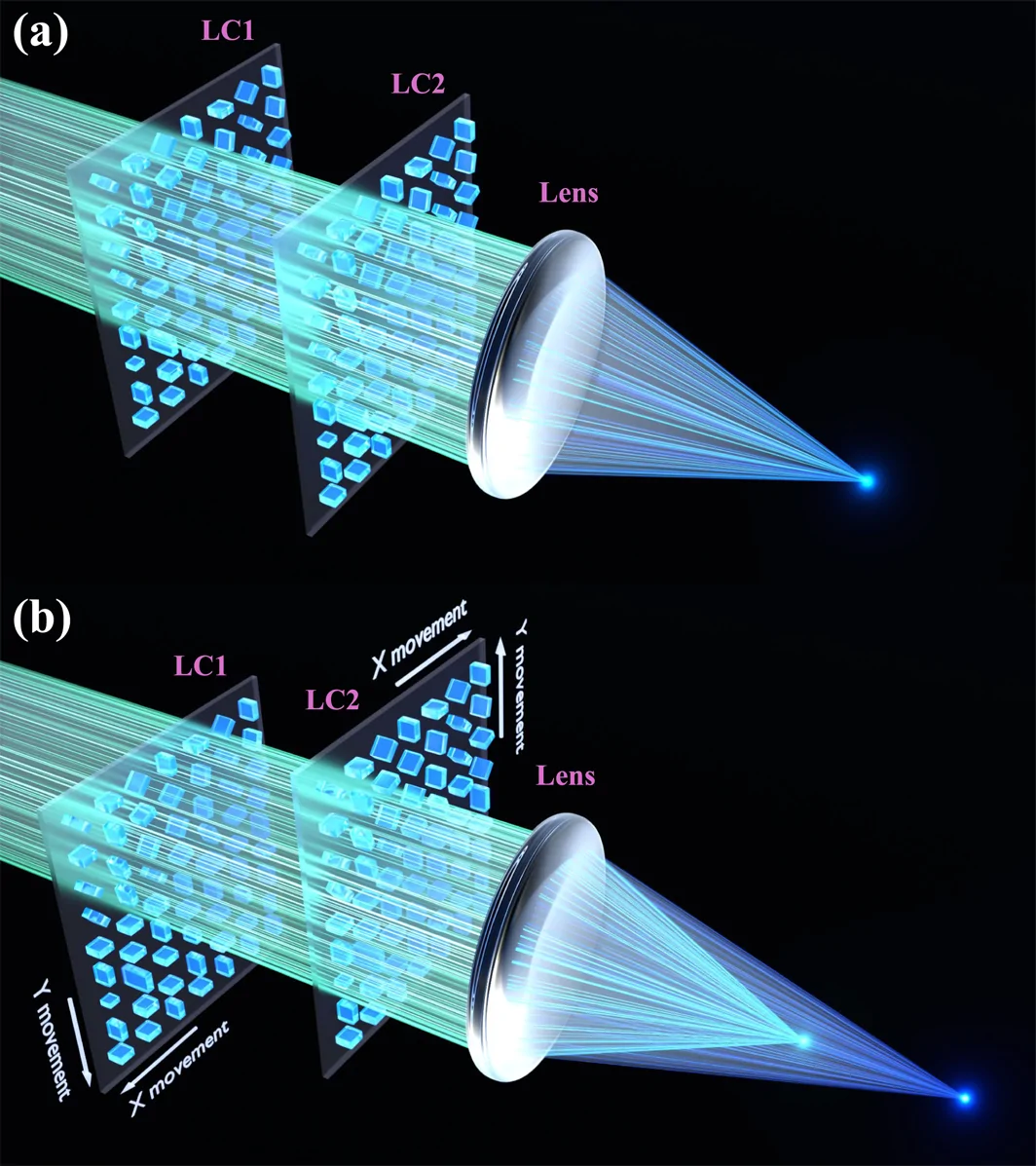
Schematic of the continuously tunable axial spin‐dependent splitting achieved by misaligning cascaded PB‐phase LC elements. (a) In the initial state, the system consists of two perfectly aligned LC elements (LC1 and LC2) cascaded with a rear convex lens. The beam converges to a single focal point. (b) When a lateral displacement is introduced between LC1 and LC2 along the diagonal direction, the LCP and RCP components focus at distinct axial positions, forming two separated focal points. In this way, the axial spin‐dependent splitting can be positive, negative, or even zero.

## 2 Principle and Design

In the theoretical analysis, a Gaussian beam is employed as the incident source. Its complex amplitude on the plane z=0 can be expressed as follows:

(1) $$E_{\text{in}}\left(x,y,0\right)=E_{G}\left(x,y\right)\left(\begin{array}{c} 1\\ \sigma i \end{array}\right),$$

where $E_{G}\left(x,y\right)=E_{0}\;\exp\left(-\frac{x^{2}+y^{2}}{\omega_{0}^{2}}\right)$ is the amplitude profile of the incident beam. Here, $E_{0}$ is the amplitude constant, $\omega_{0}$ is the beam waist radius, and σ=±1 denotes LCP and RCP light, respectively.

To achieve continuously tunable wavefront control, a cubic‐phase modulation scheme is used. The phase function of each LC element is designed as the cubic polynomial $\Phi_{0}\left(x,y\right)=\alpha \left(x^{3}+y^{3}\right)$, where α is the phase growth coefficient. Under the initial alignment, the phase distributions of the two elements cancel each other, yielding zero net phase. When a controllable transverse relative displacement vector $\delta =\left(\delta_{x},\delta_{y}\right)$ is introduced, this cancellation is disrupted, and the phase distributions $\Phi_{1}$ and $\Phi_{2}$ no longer cancel exactly. Notably, when LCP or RCP light normally illuminates the cascaded LC elements, the handedness of the incident light is flipped twice; therefore, the phases of $\Phi_{1}$ and $\Phi_{2}$ have opposite signs:

(2) $$\Phi_{1}\left(x,y\right)=\Phi_{0}\left(x-\delta_{x},y-\delta_{y}\right),$$

(3) $$\Phi_{2}\left(x,y\right)=-\Phi_{0}\left(x+\delta_{x},y+\delta_{y}\right).$$

After propagating through the cascaded LC elements, the accumulated PB phase for the left circularly polarized (LCP) component is as follows:

(4) $$\Phi_{\text{PB}}\left(x,y\right)=\Phi_{1}\left(x,y\right)+\Phi_{2}\left(x,y\right)=\alpha \left[{\left(x-\delta_{x}\right)}^{3}+{\left(y-\delta_{y}\right)}^{3}-{\left(x+\delta_{x}\right)}^{3}-{\left(y+\delta_{y}\right)}^{3}\right],$$

while for the right circularly polarized (RCP) component, the spin‐conjugate nature of the PB phase gives the opposite. Expanding this expression yields the following:

(5) $$\Phi_{\text{PB}}\left(x,y\right)=-2\alpha \left[3\left(\delta_{x}x^{2}+\delta_{y}y^{2}\right)+\left(\delta_{x}^{3}+\delta_{y}^{3}\right)\right].$$

Setting $\delta_{x}=\delta_{y}=\delta$ leads to the following equation:

(6) $$\Phi_{\text{PB}}\left(x,y\right)=-6\alpha \delta \left(x^{2}+y^{2}\right)-4\alpha \delta^{3}.$$

The constant term $-4\alpha \delta^{3}$ affects only the absolute phase and does not alter the wavefront curvature; thus it can be neglected in subsequent focusing analysis.

The system also includes a rear convex lens with phase modulation

(7) $$\Phi_{\text{lens}}\left(x,y\right)=-\frac{\pi }{\lambda f}\left(x^{2}+y^{2}\right).$$

Hence, the total phase modulation after the cascaded LC elements and the convex lens is as follows:

(8) $$\Phi_{\text{total}}\left(x,y,\sigma \right)=\sigma \Phi_{\text{PB}}\left(x,y\right)+\Phi_{\text{lens}}\left(x,y\right)=-\left(6\sigma \alpha \delta +\frac{\pi }{\lambda f}\right)\left(x^{2}+y^{2}\right),$$

where σ=±1 denote the LCP (+1) and RCP (−1) components. The outgoing field on the plane z=0 is then given by the following equation:

(9) $$E_{\text{out}}\left(x,y,0\right)=E_{\text{in}}\left(x,y,0\right)\exp\left(i\Phi_{\text{total}}\left(x,y,\sigma \right)\right).$$

To evaluate the axial spin splitting, the free‐space propagation of the field is calculated using the Fresnel diffraction integral [30]. The field $E_{\text{diff}}\left(x^{\prime },y^{\prime },z\right)$ after propagating a distance z from the z=0 plane is as follows:

(10) $$E_{\text{diff}}\left(x^{\prime },y^{\prime },z\right)=\frac{e^{\mathrm{ikz}}}{i\lambda z}\iint_{-\infty }^{\infty }E_{\text{out}}\left(x,y,0\right)\exp\left\{i\frac{\pi }{\lambda z}\left[{\left(x^{\prime }-x\right)}^{2}+{\left(y^{\prime }-y\right)}^{2}\right]\right\}dx\;dy,$$

where k=2π/λ is the wavenumber.

Substituting $E_{\text{in}}\left(x,y,0\right)$ and $\Phi_{\text{total}}\left(x,y,\sigma \right)$ into Equation (9) yields the following equation:

(11) $$E_{\text{out}}\left(x,y,0\right)=E_{0}\;\exp\left(-\frac{x^{2}+y^{2}}{\omega_{0}^{2}}\right)\exp\left[-i\left(6\sigma \alpha \delta +\frac{\pi }{\lambda f}\right)\left(x^{2}+y^{2}\right)\right]\left(\begin{array}{c} 1\\ \sigma i \end{array}\right).$$

Define $C=6\sigma \alpha \delta +\frac{\pi }{\lambda f}$, so that $E_{\text{out}}\left(x,y,0\right)=E_{0}\;\exp\left[-\left(x^{2}+y^{2}\right)\left(\frac{1}{\omega_{0}^{2}}+iC\right)\right]p$, with $p=\left(\begin{array}{c} 1\\ \sigma i \end{array}\right)$. Inserting this into Equation (10) gives us the following:

(12) $$E_{\text{diff}}\left(x^{\prime },y^{\prime },z\right)=\frac{e^{\mathrm{ikz}}}{i\lambda z}p\;E_{0}\iint_{-\infty }^{\infty }\exp\left[-\frac{x^{2}+y^{2}}{\omega_{0}^{2}}-iC\left(x^{2}+y^{2}\right)\right]\exp\left\{i\frac{\pi }{\lambda z}\left[{\left(x^{\prime }-x\right)}^{2}+{\left(y^{\prime }-y\right)}^{2}\right]\right\}dx\;dy.$$

Expanding the quadratic term $\exp\left\{i\frac{\pi }{\lambda z}\left(x^{\prime 2}+y^{\prime 2}\right)\right\}\exp\left\{i\frac{\pi }{\lambda z}\left(x^{2}+y^{2}\right)\right\}\exp\left\{-i\frac{2\pi }{\lambda z}\left(xx^{\prime }+yy^{\prime }\right)\right\}$, the integral becomes as follows:

(13) $$I=\iint \exp\left[-\left(\frac{1}{\overset{2}{\underset{0}{\omega }}}+iC-i\frac{\pi }{\lambda z}\right)\left(x^{2}+y^{2}\right)\right]\exp\left(-i\frac{2\pi }{\lambda z}\left(xx^{\prime }+yy^{\prime }\right)\right)dx\;dy.$$

Introduce the complex constant

(14) $$\gamma =\frac{1}{\omega_{0}^{2}}+i\left(C-\frac{\pi }{\lambda z}\right)=\frac{1}{\omega_{0}^{2}}+i\left(6\sigma \alpha \delta +\frac{\pi }{\lambda f}-\frac{\pi }{\lambda z}\right).$$

The two‐dimensional Gaussian integral separates into two one‐dimensional integrals:

(15) $$\int_{-\infty }^{\infty }e^{-\gamma x^{2}-i\frac{2\pi x^{\prime }}{\lambda z}x}dx=\sqrt{\frac{\pi }{\gamma }}\exp\left(-\frac{\pi^{2}x^{\prime 2}}{\gamma \lambda^{2}z^{2}}\right),$$

and similarly for y. Hence,

(16) $$I=\frac{\pi }{\gamma }\exp\left(-\frac{\pi^{2}\left(x^{\prime 2}+y^{\prime 2}\right)}{\gamma \lambda^{2}z^{2}}\right).$$

Multiplying by the prefactors, we obtain the analytical solution:

(17) $$E_{\text{diff}}\left(x^{\prime },y^{\prime },z\right)=\frac{\pi E_{0}e^{\mathrm{ikz}}}{i\lambda z\gamma }\exp\left[i\frac{\pi }{\lambda z}\left(x^{\prime 2}+y^{\prime 2}\right)-\frac{\pi^{2}\left(x^{\prime 2}+y^{\prime 2}\right)}{\gamma \lambda^{2}z^{2}}\right]\left(\begin{array}{c} 1\\ \sigma i \end{array}\right),$$

with γ defined as above. This expression describes the diffracted field of a Gaussian beam after passing through the cascaded PB phase elements and the rear convex lens, propagating a distance z in free space.

The solution in Equation (17) clearly shows that the intensity distribution remains Gaussian. Specifically, from Equation (17) we have the following equation:

(18) $$|E_{\text{diff}}{|}^{2}\propto {\left|\frac{1}{\gamma }\right|}^{2}{\left|\exp\left(-\frac{\pi^{2}\left(x^{\prime 2}+y^{\prime 2}\right)}{\gamma \lambda^{2}z^{2}}\right)\right|}^{2},$$

where γ=A+iB, and A and B are respectively defined as follows:

(19) $$A=\frac{1}{\omega_{0}^{2}},$$

(20) $$B=6\sigma \alpha \delta +\frac{\pi }{\lambda f}-\frac{\pi }{\lambda z}.$$

The modulus squared of the exponential factor is ${\left|\exp\left(-\frac{\pi^{2}r^{2}}{\gamma \lambda^{2}z^{2}}\right)\right|}^{2}=\exp\left(-2\;\frac{\pi^{2}r^{2}}{\lambda^{2}z^{2}}\;\text{Re}\;\left(\frac{1}{\gamma }\right)\right)$ with $r^{2}=x^{\prime 2}+y^{\prime 2}$. Because $\text{Re}\;\left(1/\gamma \right)=A/\left(A^{2}+B^{2}\right)$, we obtain the following equation:

(21) $$|E_{\text{diff}}{|}^{2}\propto \exp\left(-\frac{2\pi^{2}A}{A^{2}+B^{2}}\cdot \frac{r^{2}}{\lambda^{2}z^{2}}\right).$$

Comparing this with the standard Gaussian profile $\exp\left(-2r^{2}/w^{2}\right)$ yields the beam width w(z):

(22) $$w{\left(z\right)}^{2}=\frac{\lambda^{2}z^{2}\left(A^{2}+B^{2}\right)}{\pi^{2}A}.$$

Particularly, the parameter B shows that the beam width varies with propagation distance z. The beam width reaches its minimum (the focal point) when B=0, that is,

(23) $$6\sigma \alpha \delta +\frac{\pi }{\lambda f}-\frac{\pi }{\lambda z}=0.$$

Solving for z gives the total focal length of the system for a given spin state σ:

(24) $$f_{\text{total}}^{\left(\sigma \right)}=\frac{\pi }{\lambda \left(6\sigma \alpha \delta +\frac{\pi }{\lambda f}\right)},$$

which is consistent with the lens combination formula $1/f_{\text{total}}^{\left(\sigma \right)}=1/f+1/f_{\text{PB}}^{\left(\sigma \right)}$ where $f_{\text{PB}}^{\left(\sigma \right)}=\pi /\left(6\sigma \alpha \delta \lambda \right)$. The incident Gaussian beam (waist at z=0) passing through this equivalent lens will form a new waist (focus) behind the lens, generating a focusing effect. Notably, the equivalent focal length depends explicitly on σ=±1. Unless 6αδ=0, the two focal lengths are different. Therefore, the focal positions of LCP and RCP are separated along the optical axis. For example, if 6αδ>0, then $f_{\text{total}}^{\left(+1\right)}$ is positive and smaller (strong focusing), whereas $f_{\text{total}}^{\left(-1\right)}$ may be positive or negative depending on the magnitude of 6αδ relative to π/(λf). When 6αδ>π/(λf), $f_{\text{total}}^{\left(-1\right)}<0$, meaning that RCP experiences a diverging lens and its focus becomes virtual (on the left side of the lens), whereas the real focus of LCP lies on the right side. Even when both focal lengths are positive, their different values result in an axial offset between the two foci. Thus, it is theoretically confirmed that the proposed system produces a focusing effect, and more importantly, enables continuous and controllable axial spin splitting by adjusting the parameter δ.

To validate the proposed misalignment‐enabled axial spin splitting, we performed numerical simulations using the Fresnel diffraction integral method. The convex lens has a focal length $f_{\text{lens}}=300$ mm. Figure 2a1–f1 illustrate the phase reconstruction process. Each row shows, from left to right: the PB phases of LC1 and LC2, the convex lens phase, and the total cascaded phase. A lateral displacement between the two LC elements breaks the cancellation of their cubic phases, generating a residual quadratic phase that is opposite for the LCP and RCP. Combined with the lens phase, this yields tunable focusing per spin. For positive displacement ((a1),(b1)), the total phase is converging for LCP and diverging for RCP; for zero displacement ((c1),(d1)), the cubic phases cancel exactly, leaving the same pure lens phase for both spins; for negative displacement ((e1),(f1)), the signs reverse, swapping the converging/diverging roles. As shown in Figure 2a2,b2, for a positive displacement a=0.5 mm, the LCP component acquires positive focusing power, shifting its focal point to z=217 mm, whereas the RCP component exhibits negative defocusing power, elongating its focal length to z=488 mm. With no displacement (a=0 mm, Figure 2c2,d2), the cubic phase profiles of LC1 and LC2 are exactly aligned, causing the geometric phase gradients to cancel. Consequently, the PB elements contribute zero net focal power, and the system reduces to a single convex lens. Both LCP and RCP then share the same wavefront curvature and focus at z=300 mm. When a negative displacement a=−0.3 mm is applied (Figure 2e2,f2), the sign of the equivalent quadratic phase reverses. This swaps the axial order: the LCP component now focuses at z=244 mm (proximal to the lens) and the RCP at z=390 mm (distal to the lens). Thus, the splitting distance can be continuously tuned from negative through zero to positive values, and the roles of the two spin channels are dynamically interchangeable, demonstrating full reversibility. The focal‐plane intensity distributions shown in Figure 2a3–f3 reveal that under all zoom states, the beam profiles maintain circular Gaussian distributions. Meanwhile, the corresponding Stokes parameter distributions presented in Figure 2a4–f4 display clear polarization characteristics at the focal points, confirming that the system enables independent spin control while achieving large‐range continuous zooming and high beam quality.

**FIGURE 2:**
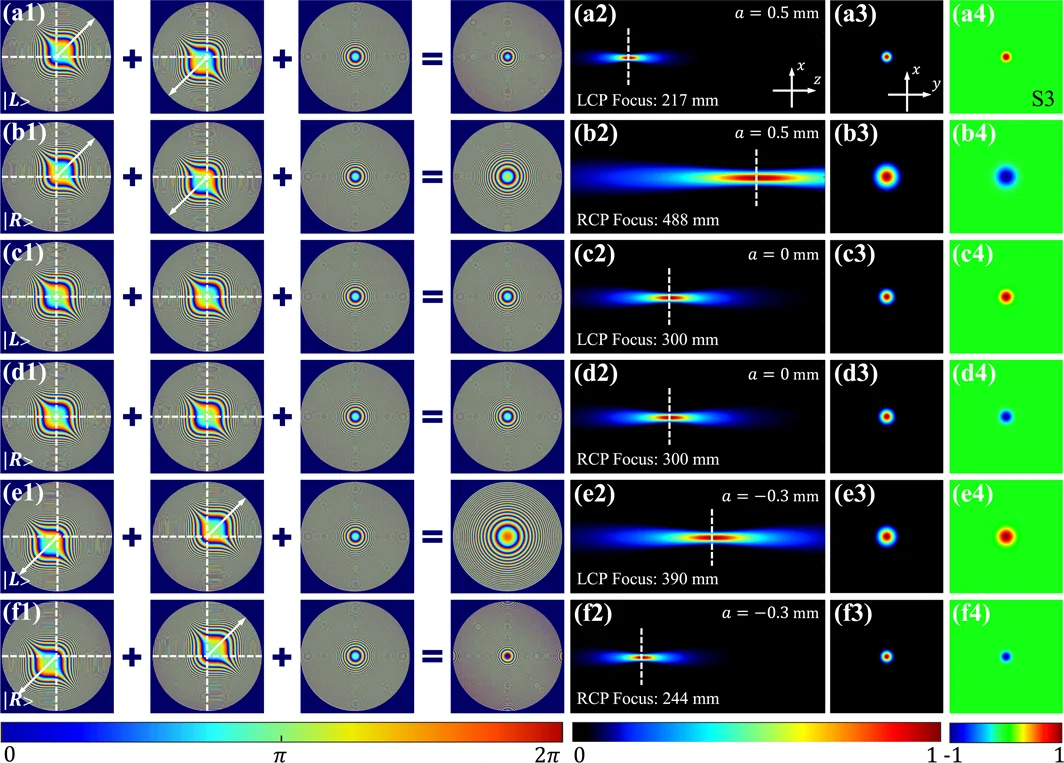
Principle of misalignment‐induced axial spin splitting. (a1–f1) Phase reconstruction process. From left to right: PB phase distributions of LC1 and LC2, the phase of the convex lens, and the total modulated phase after cascading. (a2–f2) Evolution of normalized light intensity propagation trajectories in the x–z plane. (a3–f3) Normalized intensity distributions in the x–y plane at the focal plane. (a4–f4) Stokes parameter $S_{3}$ distributions at the corresponding focal points.

## 3 Experiments and Discussions

To experimentally realize the designed cubic phase profile, two LC samples were fabricated using high‐precision laser direct writing. Transparent glass substrates were first cleaned and dried, then spin‐coated with a sulfonic azo dye (SD1) as the photoalignment layer. A laser direct writing system subsequently wrote the predetermined phase pattern point‐by‐point into this layer, where the polarization‐sensitive SD1 molecules reoriented to record the phase information. After spin‐coating a liquid crystal polymer precursor, the samples were exposed to ultraviolet light to polymerize and fix the LC molecular alignment. The resulting devices have a pixel array of 1080×1080 with a single pixel size of 8μm and a clear aperture of 8.64mm×8.64mm. To verify the half‐wave operation, we illuminated each LC element with LCP and RCP light and set the angle between the fast axis of the second QWP and the linear polarizer to +45° and −45°. The results show that the transmitted field is predominantly of the opposite handedness, confirming that the devices satisfy the approximate half‐wave retardation condition at the working wavelength. Figure 3a,d show photographs of the packaged devices. The image of LC1 under parallel polarizers (Figure 3b) exhibits high‐contrast continuous grayscale fringes, which shows the distributions of optical axes that equal twice the phase function. The magnified view in Figure 3c reveals smooth phase modulation details. For LC2 under crossed polarizers (Figure 3e), opposite‐phase fringes are observed, and even in the densely distributed regions with steep phase gradients, the fringe structure remains clear without aliasing (Figure 3f). These macroscopic observations confirm that the fabricated samples possess spatial phase modulation characteristics in excellent agreement with the theoretical design. The experimental setup for axial spin splitting is shown in Figure 3g. A 632.8 nm He–Ne laser serves as the light source. The beam intensity is modulated by a half‐wave plate (HWP) and a Glan polarizer (GLP1). A liquid crystal polarization rotator (LCPR), controlled by a function generator (FG), switches the incident polarization between LCP and RCP on a millisecond timescale. The beam then passes through LC1 and LC2 mounted on precision translation stages, followed by a fixed convex lens with focal length $f_{\text{lens}}=300$ mm. Finally, a quarter‐wave plate (QWP) and an analyzer (GLP2) are used to extract Stokes parameters, and the intensity distributions are recorded by a CMOS camera.

**FIGURE 3:**
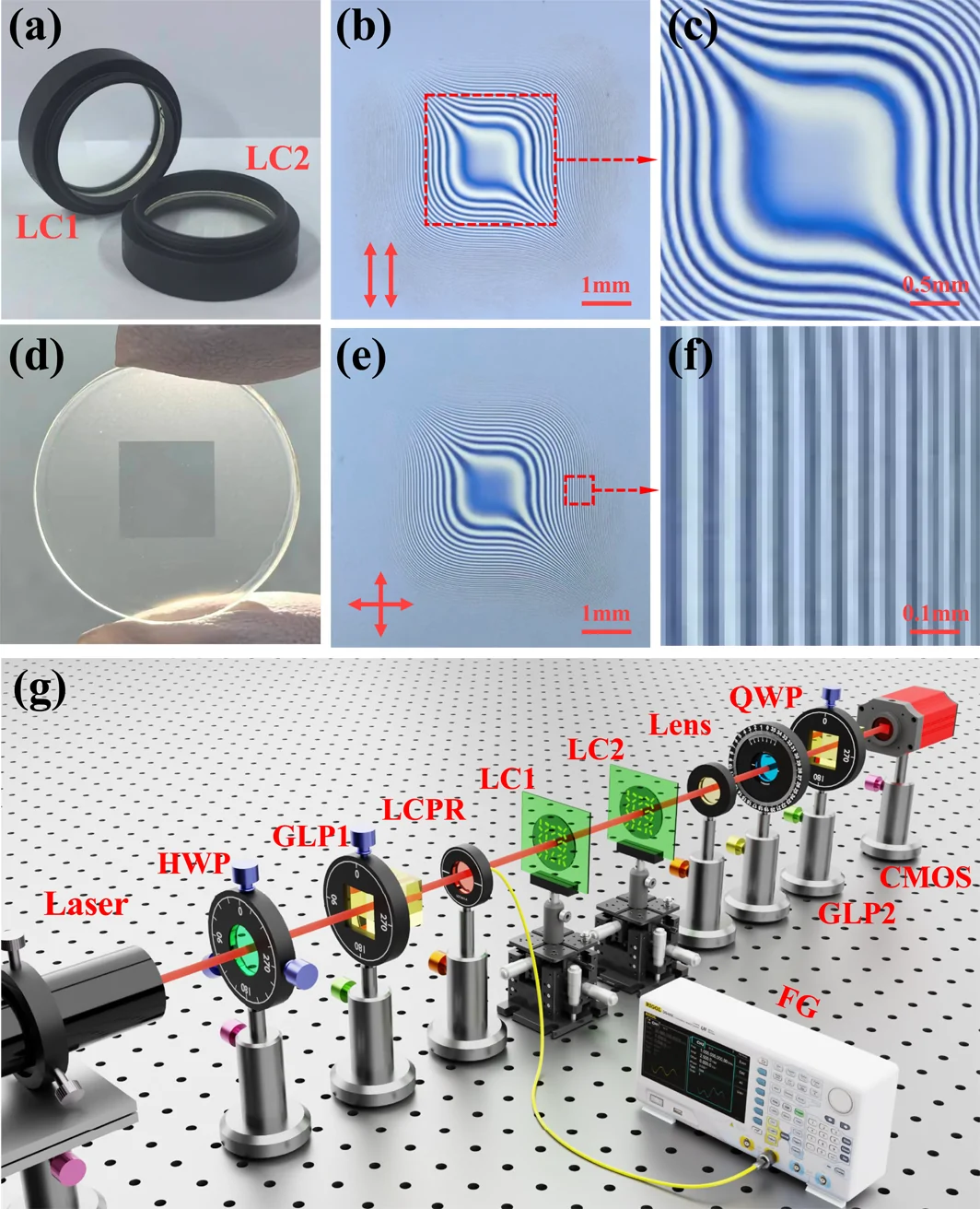
Characterization of cascaded LC PB phase elements and the experimental setup for spin splitting detection. (a) Photograph of the packaged two PB phase LC devices. (b) Parallel‐polarized image of LC1; the red double‐headed arrow indicates the polarizer orientation. (c) Magnified view of the region enclosed by the red dashed box in (b). (d) Handheld photograph of a single LC device. (e) Cross‐polarized image of LC2; the crossed red arrows indicate that the polarizer and analyzer are orthogonal. (f) Magnified view of the region enclosed by the red dashed box in (e). (g) Schematic diagram of the experimental optical setup used to probe the axial spin splitting dynamics.

In the experimental system, the incident beam was expanded threefold by lenses of $f_{\exp1}=50$ mm and $f_{\exp2}=150$ mm, then passed through the cascaded LC elements and a fixed convex lens ($f_{\text{lens}}=300$ mm). The propagation intensity in the x–z plane was reconstructed by scanning the CMOS camera with a 10 mm step. For convenience, we define $a=\sqrt{2}\delta$ as the lateral displacement magnitude. In the experiments, the displacement range of a=±0.6mm was selected as the working range rather than the upper limit. Given the effective aperture of the LC devices and the 1.05mm beam waist radius of the incident beam, the actual available displacement is approximately 4mm. For the convex lens with f=300mm, this working range already allows tuning of the LCP and RCP focal positions from 205mm to 556mm, approximately. Larger displacements would drive one spin channel toward an extremely long focal length or even a virtual focus, making precise measurement impractical. Therefore, a=±0.6mm represents a well‐balanced choice that clearly demonstrates the tunable splitting performance while maintaining high measurement reliability. To further verify that the displacement range is not limited by the phase design, we calculated the superimposed phase profiles and the corresponding focal intensity distributions for both LCP and RCP components under a convex lens with f=300mm and displacement values a=0.6,1.0,2.0,3.0,and4.0mm. The results reveal that for the LCP component, increasing a enhances the equivalent optical power and reduces the focal spot size. However, for the RCP component, the focus switches from converging to diverging when a exceeds 1.3mm. Figure 4a shows the measured focal lengths of LCP and RCP as a function of a from −0.6 mm to 0.6 mm. The experimental data (scattered points) agree well with the theoretical model (solid lines). The tuning also exhibits spin‐conjucate property: for any nonzero a, the focal length changes of LCP and RCP are equal in magnitude and opposite in sign, meeting at a=0 (black dash‐dotted line, z=300 mm). The equivalent focal power is proportional to a and its sign is spin‐dependent. Figure 4b–e map the axial intensity evolution at four representative displacements. With no displacement, as shown in Figure 4c, the PB phases cancel, the system acts as a single lens, and both spin states focus at z=300 mm. For positive displacements (a=0.3 mm and 0.5 mm), shown in Figure 4d,e, the LCP acquires positive power (proximal focus) and RCP negative power (distal focus); the splitting grows with a. For a negative displacement a=−0.5 mm, displayed in Figure 4b, the sign of the equivalent phase reverses, swapping the axial order: LCP focuses in the distance and RCP in the proximity. The system thus enables continuous, reversible tuning with dynamic interchangeability of the two spin channels. In all cases, the focal‐plane beam spots shown in the insets of Figure 4b–e maintain circular Gaussian profiles. This confirms that the displacement‐induced quadratic phase preserves wavefront fidelity across the tuning range.

**FIGURE 4:**
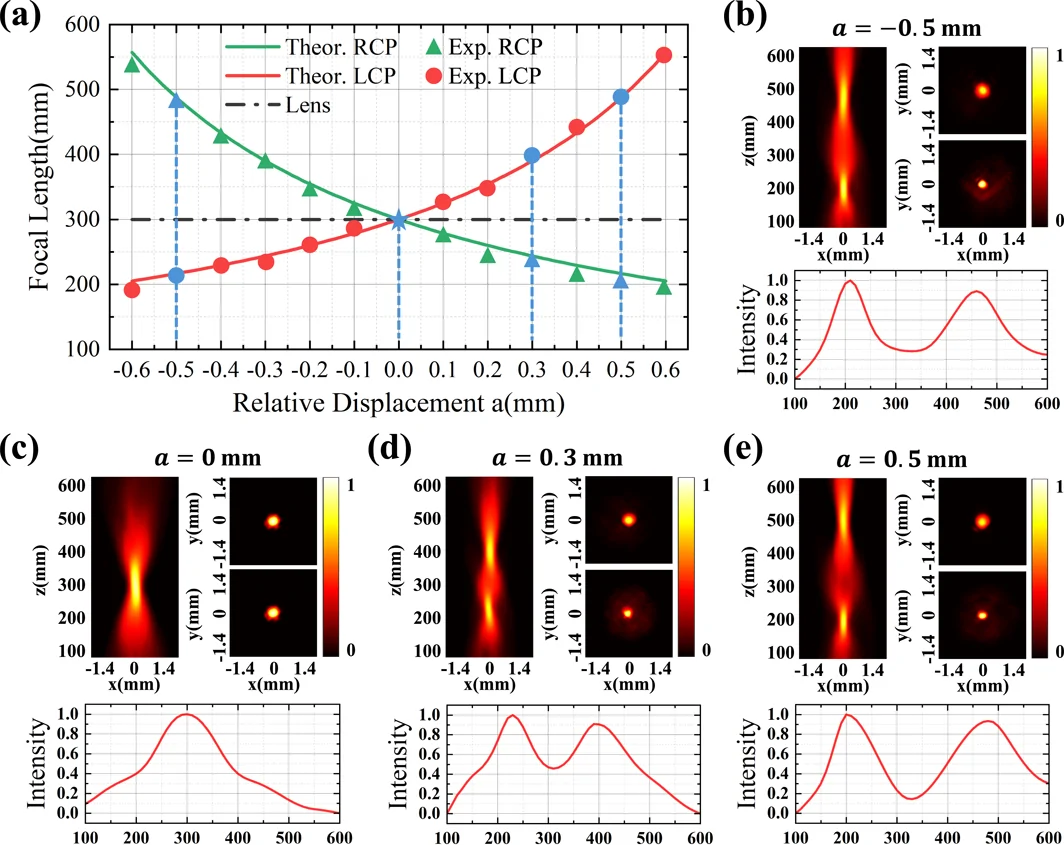
Experimentally measured continuously tunable axial spin splitting under different lateral displacements. (a) Variation of the equivalent focal length for the LCP and RCP components as a function of the relative lateral displacement, comparing theoretical predictions and experimental measurements. Red circles and green triangles represent experimentally extracted data for the LCP and RCP components, respectively; solid lines denote predictions based on the theoretical model; the black dash‐dotted line indicates the reference focal length of the convex lens; blue markers highlight the experimental data presented in the subsequent subfigures (b–e) Panoramic mapping of the free‐space optical field propagation dynamics at selected lateral displacements. Left panels show the intensity distribution in the x–z propagation plane; the two right insets display the focal spot morphology in the x–y plane at the corresponding axial positions; the bottom curves show the normalized axial intensity distribution along the z‐axis.

In addition, the tuning range of the axial spin‐dependent foci can also be controlled by varying the focal length of the convex lens. As shown in Figure 5, replacing the original lens of f=300mm with lenses of f=50mm,100mm,and500mm extends the axial spin‐splitting range from 46.4−54.2mm to 282.6−2167.9mm, approximately. Because conventional convex lenses are inexpensive and convenient for plug‐and‐play operation, this approach offers both continuous high‐precision tuning and a large tuning range for axial spin splitting. To evaluate the quality of the foci under different displacements, we performed quantitative full‐width at half‐maximum (FWHM) analysis of the spots at each focal plane. By extracting the intensity distributions and calculating the FWHM along the x and y directions, we found that at 632.8nm the FWHM values mainly lie between around 187 and 632.8μm. Similar measurements at 532nm yielded FWHM values of about 138−238μm. In both cases, the FWHM ratios are close to unity, indicating good circular symmetry and stable focusing quality.

**FIGURE 5:**
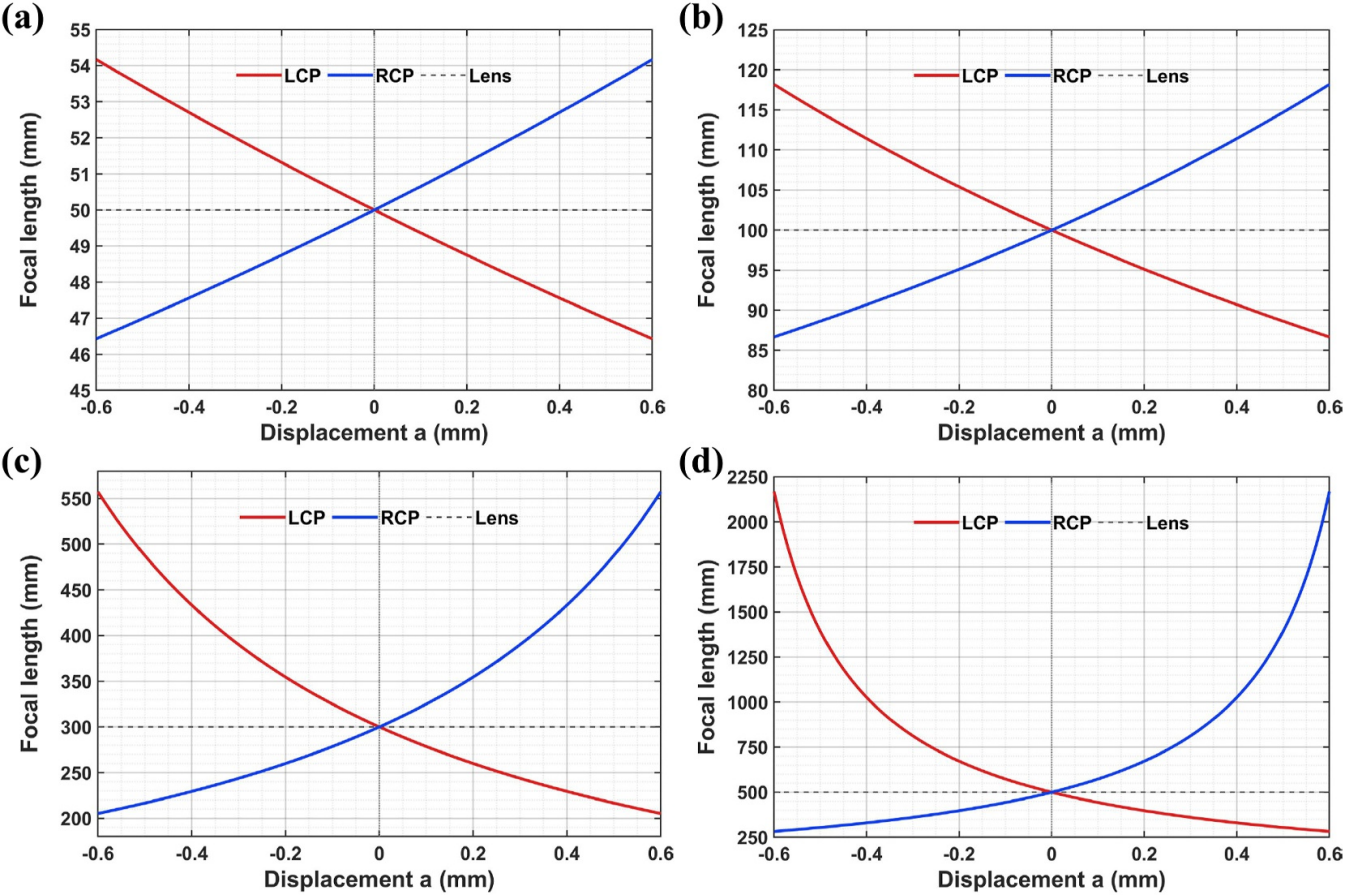
LCP and RCP focal lengths versus lateral displacement for convex lenses of (a) f=50 mm, (b) f=100 mm, (c) f=300 mm, and (d) f=500 mm, over a ±0.6 mm range. The results demonstrate that the axial spin‐splitting range can be significantly extended from approximately 46.4–54.2 mm to 282.6–2167.9 mm by simply replacing the convex lens.

To further validate the application potential of the misalignment‐enabled spin splitting mechanism for extracting three‐dimensional optical field information, we constructed a multiplane imaging test system. Considering the long imaging path imposed by the original $f_{\text{lens}}=300$ mm lens, we replaced it with a 100 mm convex lens to improve compactness and enhance collection efficiency of large‐angle scattered light. The 3× beam expansion (50 and 150 mm lenses) was retained. As illustrated in Figure 6a, a standard 1951 USAF resolution target was used, with the numeric elements “2”, “3”, and “4” positioned at different object distances u=220 mm, 180 mm, and 160 mm along the optical axis. A fixed CMOS camera served as the conjugate image plane. By adjusting the relative displacement of the cascaded LC elements, the spin‐dependent focal power is adaptively changed, enabling selective focusing on targets at different depths. According to the Gaussian imaging formula $1/u+1/v=1/f_{\text{total}}^{\left(\sigma \right)}$, where $f_{\text{total}}^{\left(\sigma \right)}$ is the spin‐dependent focal length tunable by lateral displacement, we calculated the theoretical displacement contours required for conjugate imaging of the LCP and RCP channels. Figure 6b,c show these contours, with red stars marking the three experimental operating points.

**FIGURE 6:**
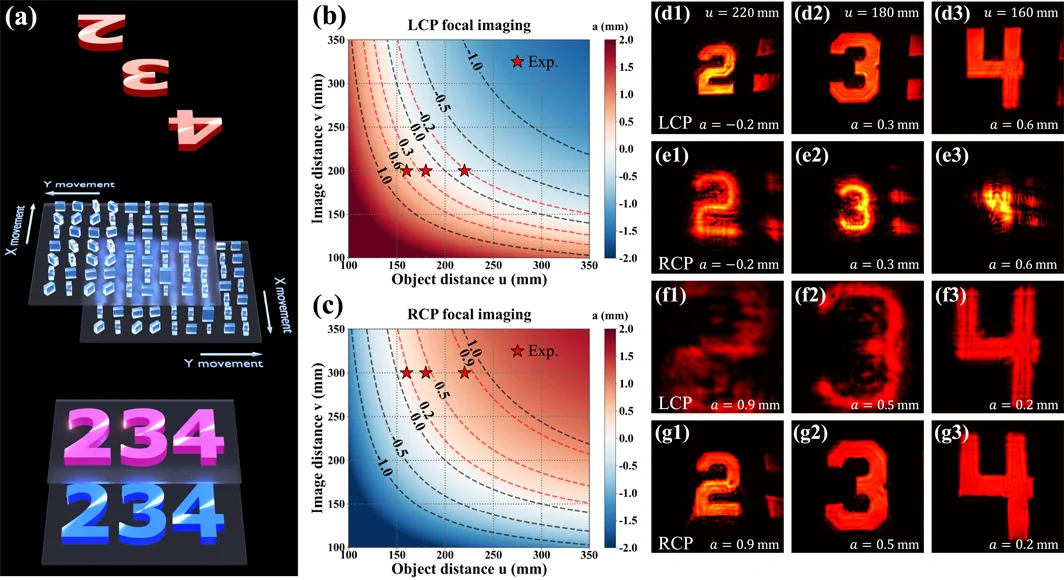
Demonstration of multiplane imaging based on continuously tunable axial spin splitting. (a) Schematic illustration of the three‐dimensional multiplane imaging system. Through the equivalent lens effect enabled by lateral displacement, object planes at different depths (the numeric targets “2,” “3,” and “4”) are selectively mapped onto a fixed detection plane. (b–c) Contour maps of the imaging conditions for LCP and RCP calculated using the Gaussian imaging formula. The color gradient indicates the precise lateral displacement required for a given object distance and image distance; red stars mark the experimental data points. (d1–d3) Sharply imaged results for the LCP channel obtained by adjusting the displacement at a fixed image distance of v=200 mm. (e1–e3) Corresponding defocused blur patterns for the RCP channel under the same image distance. (f1–f3) Defocused blur patterns for the LCP channel at a fixed distance of v=300 mm. (g1–g3) Corresponding sharply imaged results for the RCP channel obtained by adjusting the displacement under the same image distance.

With the camera fixed at v=200 mm, the displacement was scanned to a=−0.2 mm, 0.3 mm, and 0.6 mm. Under these conditions, the three targets sequentially became sharp in the LCP channel, as shown in Figure 6d1–d3, exhibiting high spatial resolution and edge sharpness. This occurs because the LCP component acquires a positive lens power proportional to the displacement, bringing the corresponding object plane into focus on the fixed detector. To more intuitively demonstrate the influence of the relative lateral displacement between the two liquid crystal devices on the imaging performance, we experimentally measured the numeral “2” with displacement a varying from 0 mm to −0.4 mm, while keeping all other measurement conditions the same as those in Figure 6d1. The corresponding dynamic process is provided as Video S1. Meanwhile, due to the spin‐conjugate nature of the PB phase, the RCP component receives an opposite lens power of equal magnitude. Consequently, its real image plane lies far behind the camera, resulting in severe defocusing and blurred images, as seen in Figure 6e1–e3. As the object distance decreases from 220 to 160 mm, the image size increases significantly, consistent with the transverse magnification rule M=v/u, confirming that the system preserves geometric imaging fidelity.

When the camera was moved to v=300 mm, as shown in Figure 6f,g, the roles of the two spin channels reversed. With displacements set to a=0.9 mm, 0.5 mm, and 0.2 mm, the RCP channel produced clear images of the three targets (Figure 6g1–g3), whereas the LCP channel became blurred (Figure 6f1–f3). The same magnification trend with decreasing u was observed. This reciprocal behavior is a direct manifestation of the spin‐antisymmetric tuning characteristic established in Figure 4a: for a given displacement magnitude a, the focal lengths of LCP and RCP deviate equally in opposite directions from the baseline f=300 mm. When a>0, LCP focuses at a shorter distance (proximal) and RCP at a longer distance (distal); when a<0, the assignment swaps. Consequently, by simply changing the sign of the lateral misalignment, the system dynamically reassigns which spin channel delivers sharp imaging on a fixed detector plane. Notably, this depth switching is achieved without any axial motion of optical components or the sample. The displacement is applied purely in the transverse plane (perpendicular to the optical axis). All elements remain stationary along the z‐direction, keeping the total optical path length constant and avoiding realignment of downstream optics. Moreover, because the system generates two foci at distinct axial positions, placing the camera at one focal plane yields a sharp image from the corresponding spin channel while the other spin channel produces a defocused background. Thus, a single exposure captures information from two different depth planes in parallel, one in focus, the other as a controlled blur, enabling depth‐resolved imaging without axial scanning. This highlights the system's potential for high‐speed, compact, and spin‐dependent three‐dimensional imaging.

## 4 Conclusions

We have demonstrated continuously tunable axial spin‐dependent splitting in cascaded LC PB phase elements via controlled lateral misalignment. The relative displacement generates a spin‐dependent quadratic phase, enabling linear tuning of the focal lengths for LCP and RCP components over a range of ±0.6 mm with opposite signs. Experimental results agree well with theoretical predictions, and the system maintains high imaging fidelity across the tuning range. A key feature is the simultaneous generation of two distinct foci at different axial positions for the two spin channels, allowing parallel depth acquisition; reversing the displacement sign dynamically interchanges the proximal and distal foci. Leveraging this capability, we further demonstrate multiplane imaging. This combination of compact hardware and parallel acquisition offers a practical alternative to conventional axial‐scanning methods, particularly for applications that demand fast depth switching or simple mechanical design.

## Author Contributions

**Yougang Ke:** conceptualization, methodology, funding acquisition, supervision. **Xinhuai Gu:** data curation, investigation, visualization, software. **Linzhou Zeng:** investigation, writing – review and editing, writing – original draft, supervision. **Zhangming Hong:** visualization. **Jingqing Yu:** validation. **Yingqun Xiao:** resources, project administration. **Xinxing Zhou:** writing – review and editing. **Jin Li:** writing – review and editing.

## Supporting information


**Video S1:** Real‐time evolution of the image of target “2” as the lateral displacement between the two LC elements is continuously varied from 0 to −0.4 mm. The imaging quality first improves, reaching its best focus at −0.2 mm, and then gradually degrades as the displacement increases further. Measurement conditions correspond to those in Figure 6d1 of the main text.

## Data Availability

The data that support the findings of this study are available from the corresponding author upon reasonable request.

## References

[1] M. Onoda , S. Murakami , and N. Nagaosa , “Hall Effect of Light,” Physical Review Letters 93, no. 8 (2004): 083901, 10.1103/physrevlett.93.083901. 15447185

[2] X. Yin , Z. Ye , J. Rho , Y. Wang , and X. Zhang , “Photonic Spin Hall Effect at Metasurfaces,” Science 339, no. 6126 (2013): 1405–1407, 10.1126/science.1231758. 23520105

[3] K. Y. Bliokh , F. J. Rodríguez‐Fortuño , F. Nori , and A. V. Zayats , “Spin–Orbit Interactions of Light,” Nature Photonics 9, no. 12 (2015): 796–808, 10.1038/nphoton.2015.201.

[4] O. Hosten and P. Kwiat , “Observation of the Spin Hall Effect of Light via Weak Measurements,” Science 319, no. 5864 (2008): 787–790, 10.1126/science.1152697. 18187623

[5] N. Yu , P. Genevet , M. A. Kats , et al., “Light Propagation With Phase Discontinuities: Generalized Laws of Reflection and Refraction,” Science 334, no. 6054 (2011): 333–337, 10.1126/science.1210713. 21885733

[6] H. Bao , F. Zhang , M. Pu , et al., “Field‐Driven Inverse Design of High‐Performance Polarization‐Multiplexed Meta‐Devices,” Laser & Photonics Reviews 18, no. 8 (2024): 2301158, 10.1002/lpor.202301158.

[7] X. Shi , H. Lin , T. Liu , et al., “Polarization‐Controlled Non‐Hermitian Metasurfaces for Ultra‐Sensitive Terahertz Sensing,” Laser & Photonics Reviews 19, no. 14 (2025): 2500172, 10.1002/lpor.202500172.

[8] H. He , D. Yu , Y. Gu , et al., “Dynamically Polarization‐Dispersion‐Modulated LC‐Integrated Metasurfaces for Hybrid‐Dimensions Encryption,” Laser & Photonics Reviews 19, no. 20 (2025): e00564, 10.1002/lpor.202500564.

[9] D. Lin , P. Fan , E. Hasman , and M. L. Brongersma , “Dielectric Gradient Metasurface Optical Elements,” Science 345, no. 6194 (2014): 298–302, 10.1126/science.1253213. 25035488

[10] F. Zhang , H. Bao , M. Pu , et al., “Dispersion‐Engineered Spin Photonics Based on Folded‐Path Metasurfaces,” Light: Science & Applications 14, no. 1 (2025): 198, 10.1038/s41377-025-01850-w. PMC12084559 40379638

[11] Q. Fan , P. Lin , L. Tan , et al., “Non‐Invasive and Fully Two‐Dimensional Quantitative Visualization of Transparent Flow Fields Enabled by Photonic Spin‐Decoupled Metasurfaces,” Light: Science & Applications 14, no. 1 (2025): 113, 10.1038/s41377-025-01793-2. PMC11882825 40044662

[12] S. Chen , J. Chen , T. Xia , et al., “Optical Vortices in Communication Systems: Mode (de)Modulation, Processing, and Transmission,” Advanced Photonics 7, no. 4 (2025): 044001, 10.1117/1.AP.7.4.044001.

[13] S. Zhou , J. Bian , P. Chen , et al., “Polarization‐Dispersive Imaging Spectrometer for Scattering Circular Dichroism Spectroscopy of Single Chiral Nanostructures,” Light: Science & Applications 11, no. 1 (2022): 64, 10.1038/s41377-022-00755-2. PMC8933428 35304873

[14] J. Zhou , H. Qian , C. F. Chen , et al., “Optical Edge Detection Based on High‐Efficiency Dielectric Metasurface,” Proceedings of the National Academy of Sciences 116, no. 23 (2019): 11137–11140, 10.1073/pnas.1820636116. PMC6561260 31101711

[15] H. Guo , C. Xu , Q. Xiong , X. Zhang , J. Wang , and X. Qiu , “Optical‐Vortex‐Convolution‐Enhanced Neural Network,” Laser & Photonics Reviews 19, no. 23 (2025): e00927, 10.1002/lpor.202500927.

[16] K. Zeng , Y. Ke , Z. Hong , L. Zeng , and X. Zhou , “In‐Situ and Ex‐Situ Twisted Bilayer Liquid Crystal Computing Platform for Reconfigurable Image Processing,” Opto‐Electronic Advances 8, no. 12 (2025): 250226, 10.29026/oea.2025.250226.

[17] X. Li , Y. Kim , Y. Jeon , et al., “Parallel Bright‐Field and Multi‐Order Edge Imaging via Wide Field‐of‐View Trichannel Metalens,” Opto‐Electronic Advances 9, no. 7 (2026): 250306, 10.29026/oea.2026.250306.

[18] Y. He , Y. Zhu , H. Luo , et al., “Optical Topological Differentiation Cryptography,” Laser & Photonics Reviews 20, no. 11 (2026): e02957, 10.1002/lpor.202502957.

[19] Y. Ke , Y. Bian , Q. Tang , et al., “Rotational Photonic Spin Hall Effect,” Nanophotonics 12, no. 23 (2023): 4361–4373, 10.1515/nanoph-2023-0559. PMC11501681 39634714

[20] S. Liu , S. Qi , Y. Li , B. Wei , and J. Zhao , “Controllable Oscillated Spin Hall Effect of Bessel Beam Realized by Liquid Crystal Pancharatnam‐Berry Phase Elements,” Light: Science & Applications 11, no. 1 (2022): 219, 10.1038/s41377-022-00888-4. PMC9276670 35821002

[21] J. Zhou , F. Tian , J. Hu , et al., “Eagle‐Eye Inspired Meta‐Device for Phase Imaging,” Advanced Materials 36, no. 32 (2024): 2402751, 10.1002/adma.202402751. 38816897

[22] L. Li , M. Liao , Y. Zhang , et al., “Monocular Complex Amplitude Imaging via a Polarization‐Multiplexed Liquid‐Crystal‐Lens‐Informed Fourier Neural Network,” National Science Review 13, no. 2 (2026): nwaf561, 10.1093/nsr/nwaf561. PMC12839537 41608046

[23] Y. Ke , Y. Liu , J. Zhou , H. Luo , and S. Wen , “Optical Integration of Pancharatnam‐Berry Phase Lens and Dynamical Phase Lens,” Applied Physics Letters 108, no. 10 (2016): 101102, 10.1063/1.4943403.

[24] T. Badloe , I. Kim , Y. Kim , J. Kim , and J. Rho , “Electrically Tunable Bifocal Metalens With Diffraction‐Limited Focusing and Imaging at Visible Wavelengths,” Advanced Science 8, no. 21 (2021): 2102646, 10.1002/advs.202102646. PMC8564427 34486242

[25] B. Xu , W. Wei , P. Tang , et al., “A Multi‐Foci Sparse‐Aperture Metalens,” Advanced Science 11, no. 19 (2024): 2309648, 10.1002/advs.202309648. PMC11109648 38483885

[26] R. Lin , J. Yao , C. Chen , et al., “Varifocal Meta‐Lens for Multifunctional Focusing and Imaging,” Advanced Science 12, no. 45 (2025): e14015, 10.1002/advs.202514015. PMC12677681 40977360

[27] H. Li , C. Zhao , W. Xu , et al., “Twisted Bilayer Meta‐Device for On‐Demand Terahertz Polarization Filtering,” Photonics Research 13, no. 5 (2025): 1116, 10.1364/prj.554358.

[28] C. L. Zheng , P. N. Ni , Y. Y. Xie , and P. Genevet , “On‐Chip Light Control of Semiconductor Optoelectronic Devices Using Integrated Metasurfaces,” Opto‐Electronic Advances 8, no. 1 (2025): 240159, 10.29026/oea.2025.240159.

[29] C. L. Zheng , W. J. Dou , K. X. Li , et al., “Broadband Vectorial Holography From the Visible to the Deep Ultraviolet Region Using Diamond Metasurfaces,” ACS Nano 20, no. 17 (2026): 13235–13244, 10.1021/acsnano.6c02354. 42012225

[30] J. W. Goodman , Introduction to Fourier Optics (Roberts and Company Publishers, 2005).

